# Direct measurement of the mechanical properties of a chromatin analog and the epigenetic effects of *para*-sulphonato-calix[4]arene

**DOI:** 10.1038/s41598-019-42267-x

**Published:** 2019-04-09

**Authors:** Yannick Tauran, Momoko Kumemura, Mehmet C. Tarhan, Grégoire Perret, Florent Perret, Laurent Jalabert, Dominique Collard, Hiroyuki Fujita, Anthony W. Coleman

**Affiliations:** 1 LMI CNRS UMR 5615, Université Lyon 1, Villeurbanne, 69622 France; 2 LIMMS/CNRS-IIS UMI 2820, Institute of Industrial Science, The University of Tokyo, Tokyo, 153-8505 Japan; 3 CIRMM, Institute of Industrial Science, The University of Tokyo, Tokyo, 153-8505 Japan; 4 Graduate School of Life Science and Systems Engineering, Kyushu Institute of Technology, Fukuoka, 808-0196 Japan; 5 Univ. Lille, CNRS, Centrale Lille, ISEN, Univ. Valenciennes, UMR 8520-IEMN, Lille, F59000 France; 6 CNRS/IIS/COL/Lille 1 SMMiL-E project, 59046 Lille Cedex, France; 7 ICBMS, CNRS UMR 5246, Université Lyon 1, Villeurbanne, 69622 France

## Abstract

By means of Silicon Nano Tweezers (SNTs) the effects on the mechanical properties of λ-phage DNA during interaction with calf thymus nucleosome to form an artificial chromatin analog were measured. At a concentration of 100 nM, a nucleosome solution induced a strong stiffening effect on DNA (1.1 N m^−1^). This can be compared to the effects of the histone proteins, H1, H2A, H3 where no changes in the mechanical properties of DNA were observed and the complex of the H3/H4 proteins where a smaller increase in the stiffness is observed (0.2 N m^−1^). *Para*-sulphonato-calix[4]arene, SC4, known for epigenetic activity by interacting specifically with the lysine groups of histone proteins, was studied for its effect on an artificial chromatin. Using a microfluidic SNT device, SC4 was titrated against the artificial chromatin, at a concentration of 1 mM in SC4 a considerable increase in stiffness, 15 N m^−1^, was observed. Simultaneously optical microscopy showed a physical change in the DNA structure between the tips of the SNT device. Electronic and Atomic Force microscopy confirmed this structural re-arrangement. Negative control experiments confirmed that these mechanical and physical effects were induced neither by the acidity of SC4 nor through nonspecific interactions of SC4 on DNA.

## Introduction

With the recent and better knowledge on how gene regulation occurs and after intense debate in the academic community^1^, epigenetics has been commonly defined as “the study of changes in gene function that are mitotically and/or meiotically heritable and that do not entail a change in DNA sequence”^2^.

Over the past years, epigenetics has been shown to play a central role in many different key functions at the cellular level, including differentiation, replication, and gene transcription^3^. In a larger context, its contribution in physiological disorders has been revealed in cardio vascular illnesses, mental disorders and various cancers^4^.

Despite its inheritable character, epigenetics paradoxically possesses a reversible nature^5^ that can be illustrated through its two main covalent modifications either directly on DNA with the methylation of cytosine nucleic bases or indirectly with various post translational modifications on proteins interacting with DNA such as histone proteins or other proteins called writers, erasers or readers^6^. New therapeutic treatments are expected to target and reverse these modifications by inactivating the enzymes responsible for the deregulation of specific genes^7^. Some inhibitors have been already approved by FDA, such as Azacitidine and Decitabine as DNA methyltransferase inhibitors for the treatment of myelodysplastic syndrome^8^ and Vorinostat and Romidepsin as inhibitors of histone deacetylase for the treatment of peripheral T-cell lymphoma^9^. A new class of DNA artificial binders that directly block the methyllysine reading functions of reader enzymes in charge of conveying the methylation signal downstream has recently arisen as promising epigenetic drugs^10^.

**Figure 1:**
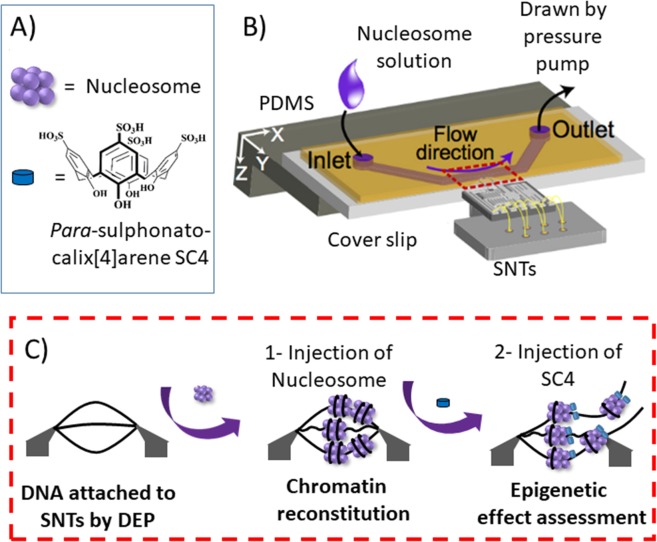
Schematic representation of the set up for reconstituting chromatin and make mechanical epigenetic assay. (**A**) Scheme representation of histone and *para*-sulphonato-calix[4]arene **SC4** with its given structure. (**B**) Setup for the SNT device immersed in the lateral opening of the channel of the microfluidic device. (**C**) Entering in the channel via the side opening, the captured DNA bundle between SNTs tips interact first with nucleosome for reconstituting chromatin and then assess the mechanical stress of epigenetic drugs.

*Para*-sulphonato-calix[4]arene, **SC4** (Fig. 1A) has been particularly efficient in inhibiting reading functions, with a high affinity *in vitro* with a Kd of 5 µM^11^. At the cellular level **SC4** blocks the interaction between the protein reader Plant Homodomain and the trimethylated lysine 4 of Histone H3 methylated^12^. The anionic calix[n]arenes have been widely studied for their ability to complex amino-acids^13^, peptides^14^, in particular those containing basic amino acids^15^ and proteins^16,17^. A considerable body of structural knowledge on the structure of calix[n]arenes has been in depth by Suwinska^18^ and the structures of their complexes with proteins have been reported by Crowley and Hof^19,20^. The cellular and *in vivo* behaviour of these supra-molecules has been recently reported by ourselves^21^ and most importantly a lack of *in vivo* toxicity observed in animal studies^22^. Such behaviour singles out the anionic calix[n]arenes and especially the *para*-sulphonato-calix[n]arene derivatives as preferential candidates for use as supramolecular epigenetics agents.

For reversing or preventing a specific epigenetic modification in order to re-establish the tissue homeostasis, full understanding of how the DNA and its associated proteins interact with their environment while maintaining their normal cellular functions, is required. Such understanding requires suitable methods to study the properties of DNA during its rearrangement in the nucleus, (folding-unfolding) and how the mechanical properties of DNA can weigh on the nucleosome complexes of Chromatin^23^. The nucleosome is formed by the deposition of an H3/H4 tetramer (in fact a dimer of H3/H4 dimeric duplex complexes) on DNA followed by two sets H2A/H2B dimers. This forms an octameric complex consisting of eight globular proteins, with the two amino-terminal domains of the H3 proteins pointing outward^3^.

Recently, the development of Next Generation Sequencing technologies combined with Chromatin Human Immuno Precipitation^24^ or Methyl Sequencing techniques^25^ has made it possible to accurately map the human genome and identify the sites on DNA at which occur specific epigenetic modifications. Even if these new techniques are highly significant, they remain very costly and can be qualified as ‘static’, that is, not able to describe the ‘dynamic’ behaviour of the chromatin that regulates the gene expression^26^.

New technologies coming from micro and nanotechnologies have recently been developed for addressing this ‘dynamic’ issue^27^ such as DNA curtains^28^, or single molecule force spectroscopies^29^. However, even if the DNA curtains methodology offers a molecular crowded environment more biologically significant than single molecule force spectroscopy for understanding how proteins interact, displace and reorganize chromatin^30^, it suffers from different drawbacks including; costly equipment (with Total Internal Reflection Fluorescence microscope) and a complex and long protocol prior to experiments (liposome preparation, labelling proteins and DNA with fluorescent markers)^31^. On the other hand, among single molecule force spectroscopies, magnetic tweezers have shown to possess a high enough force sensitivity (10^−2^ pN)^32^ compared to optical tweezers (1 pN)^33^ for stretching and sensing the different conformations of a single chromatin fibre^34^. Despite the obvious interest to understand how epigenetic influence the coupled nucleosomes along DNA, there is also a need to understand the activity and mechanism of the chromatin at a higher level of compaction, closer to the nuclear environment (higher number of chromatin fibres complexed with a larger of DNA associated proteins)^35^. Besides, on a practical point of view, techniques should become cheaper and easier to perform in order to be spread and used routinely in biological laboratories.

Recently, Silicon Nano Tweezers (SNTs) have been shown to be a cost effective and rapid methodology for real-time monitoring of biomolecular interactions on DNA^36^, even under extreme conditions^37^. The aluminium coated and opposed tips can trap a bundle of label-free DNA at high molecular density (0.8 × 10^6^–0.8 × 10^7^ bp µm^−3^) within a minute using dielectrophoresis DEP (see Materials and Methods). Software control allows accurate insertion in the lateral opening of the microfluidic device (Fig. 1B). This allows injection of a series of molecular solutes in order to assemble molecular complexes^38^ and/or to determine the mechanical effects as a function of the concentration of a given substrate on DNA packing and rearrangement in terms of stiffness and viscous losses changes^39^.

Here, we propose for the first time, the use of Silicon Nano Tweezers combined with a microfluidic device in order to reconstitute a nucleosome and measure directly in real time how such a complex or its constituent parts can affect the mechanical properties of DNA (Fig. 1C). In a second step the effects of the supramolecular epigenetic agent *para*-sulphonato-calix[4]arene on the nucleosome-DNA body are studied.

## Results

Chromatin reconstitution *in vitro* has been previously described in the literature by two main methods depending on the use of ATPase enzyme for assembling the five types of histone proteins in a nucleosome along the DNA^40^. While the ATPase dependent method produces a periodic nucleosome array with any DNA sequence of indefinite length, the ATPase-independent reconstitution of nucleosomes is random along the DNA position^41^. In fact, we selected this latter method as it presents the advantage of producing a pure chromatin (consisting of only purified core histone proteins and DNA) unencumbered by histone chaperones or other biomolecules that could interfere with the subsequent epigenetic assessments.

Therefore, in order to reconstitute nucleosome on our SNTs, DNA was firmly attached, using DEP between the tips of the SNTs and then immersed in aqueous solutions with containing increasing concentrations of nucleosomes (Fig. 1). Since nucleosome keeps its structure in water and the counter ions along the DNA diffuse in solution, nucleosome complex reconstitution is favoured. DNA curtain experiments have already characterized nucleosome attachment along λ phage DNA under crowded molecular environments^31^. However, conventional methods for characterizing the chromatin between the tweezer tips such as micrococcal nuclease digestion assay, gel mobility shift analysis or Atomic Force Microscopy were not appropriate at this low DNA concentration level. Thus, the characterization of the nucleosome formation has been performed by the mechanical sensing of SNTs itself.

Increasing concentrations of histone proteins H2A, H3, a 1:1 mix of histone proteins H3 and H4 and, finally, nucleosome, previously purified from calf thymus, were in separate experiments titrated against purified λ phage DNA trapped on SNTs. The highest concentration of each histone protein or nucleosome solution was selected in view of the solubility of the protein system. All experiments have been performed in a duplicate using two different DNA bundles.

To establish the specific mechanical packing of nucleosome along the DNA, we compared it with single or double core histone types as negative controls. Indeed, histone proteins possess a part of the physico-chemical properties of the nucleosome, without its ability to be wrapped by DNA.

**Figure 2:**
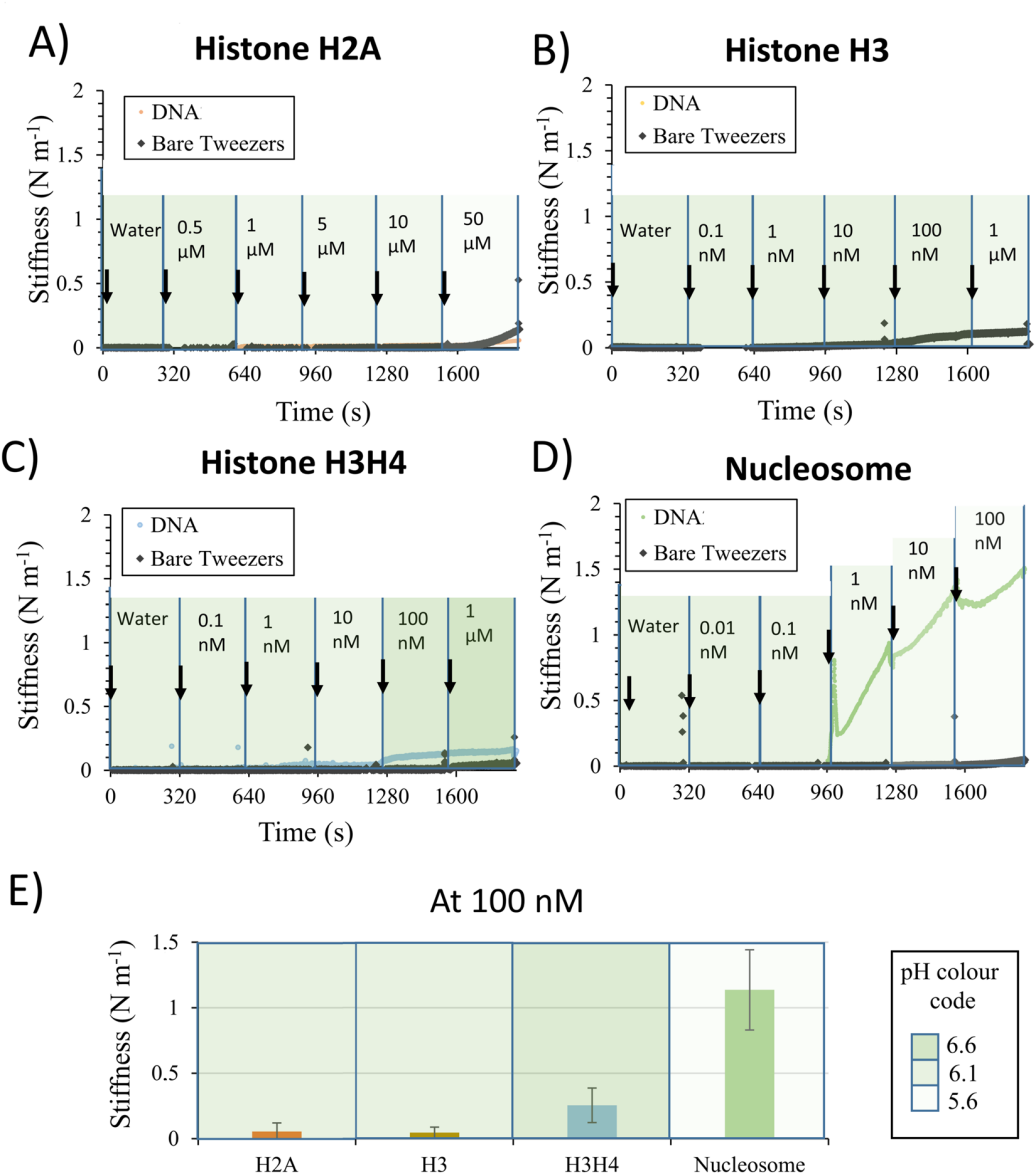
Stiffening effect on DNA of nucleosome compared to histone proteins types for reconstituting chromatin. Real time monitoring of the stiffness on SNTs by injecting increasing concentration of (**A**) histone protein H2A, (**B**) histone protein H3, (**C**) histone protein duplex H3/H4, and (**D**) nucleosome. (**E**) Histogram of stiffness average after 4 minutes of incubation of either histone protein H2A, or histone protein H3, or histone protein duplex H3/H4, or nucleosome at 100 nM. pH value of each sample is represented by the universal color code of pH paper and indicated in the inset (green for neutral pH, red for acidic pH). pH data from all samples are given in Table S1.

As expected, no mechanical effects were observed when either increasing concentrations of histone protein H2A (up to 50 µM) or H3 (up to 1 µM) interacted with the trapped DNA bundle (Fig. 2A,B). The H3/H4 histone protein duplex which is known to bind DNA under the form of a tetrameric complex (2 histone proteins H3 and 2 histone proteins H4) but which is unable to pack DNA in the absence of the complementary histone protein complex H2A and H2B^42^ shows a slight, but significant, increase in stiffness (0.2 N m^−1^) at 1 µM (Fig. 2C). Finally, a much stronger mechanical stiffening effect occurred for DNA when the nucleosome (1.1 N m^−1^) was injected at 100 nM (Fig. 2D–E and Supplementary Fig. S1). This specific mechanical effect on DNA, shows that the nucleosome is able to interact with DNA, thus reconstituting a chromatin, and generating the expected changes in the mechanical properties of DNA induced by packing around the nucleosome.

After injecting the nucleosome solution into the microfluidic device (in presence of the SNTs with DNA between the tips), the system was incubated during 5 minutes to form a chromatin type complex, the solution was then exchanged with water to remove any nucleosome unattached to the DNA bundle. Chromatin consisting of nucleosome and DNA has been shown to be stable in water^43^. Once the chromatin is reconstituted, the mechanical effect of molecule with epigenetic activity have been assessed by titration. Since common buffer molecules have been shown to interact with *para*-sulphonato-calix-[4]-arene SC4^44^, all biomolecules assays have been performed in water. Two different control experiments (for pH with nitric acid and for macrocyclic effects with *para*-hydoxybenzenesulfonic acid) have been included. The results confirm that neither the mechanical nor the physical effects that we have observed are induced by pH or simple units of the SC4 macrocycle. In a final control the full titration on nucleosome was undertaken at a buffer concentration of 50 mM, this being both the intracellular sodium concentration and the value used by Hof, the results show that the SNT functions normally at this salt concentration and the results accord with the full set of titrations, Supplementary Fig. S2.

**Figure 3:**
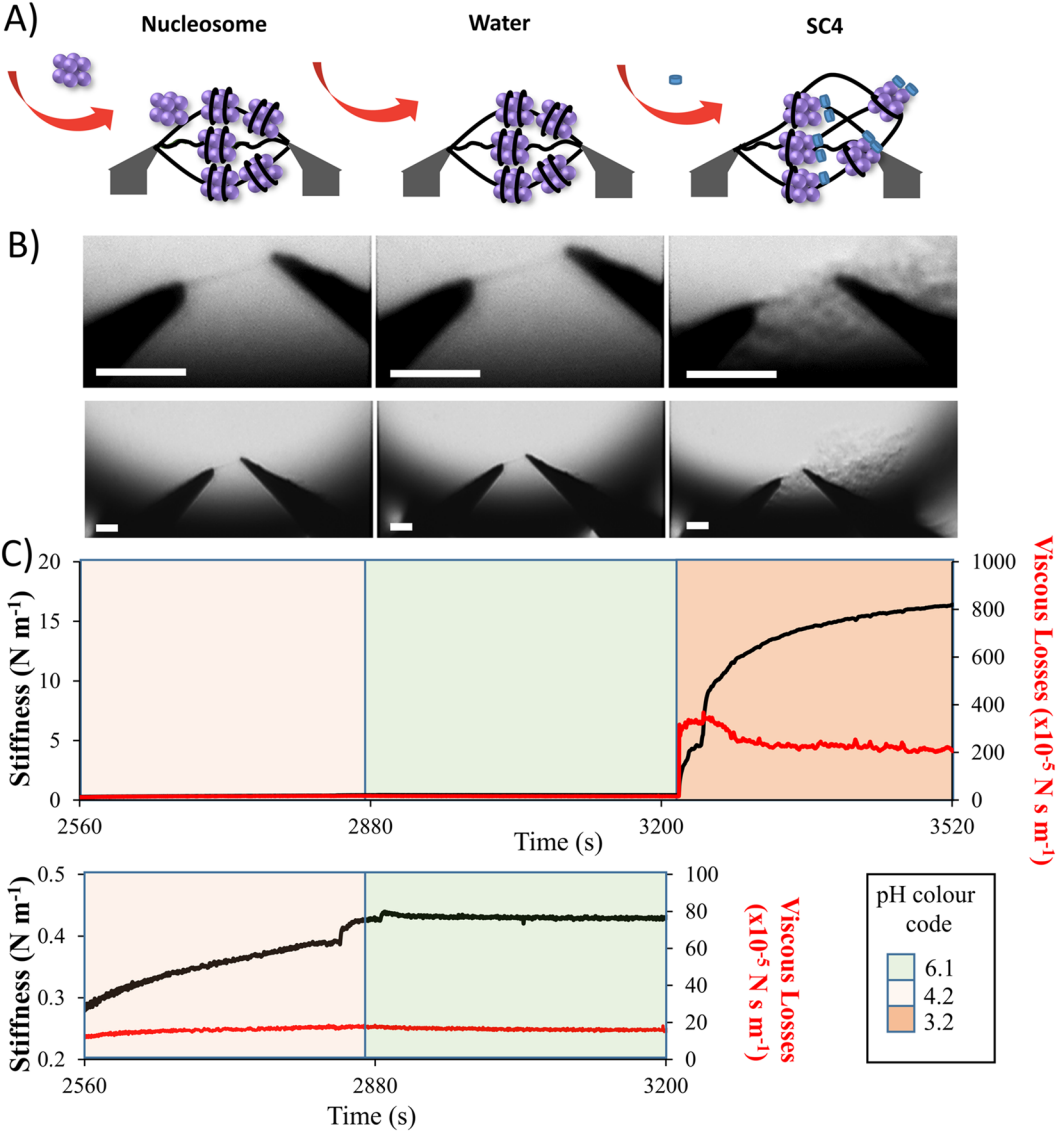
Mechanical and Physical effects of SC4 on reconstituted chromatin. (**A**) Schematic representation of the injection of nucleosome on the bundle of DNA trapped by SNT tips. (**B**) Optical microscopy images at high (images at the top) and low (images at the bottom) magnification of DNA trapped by SNT in the microfluidic device after injection (from left to right) of (100 µM) nucleosome, water and (1 mM) **SC4**. White scale bars represent 10 µm. (**C**) Real time monitoring of the stiffness and viscous losses of DNA as the graph at the top. At first nucleosome was injected (0–320 s), which was followed by the consecutive injections of water (320–640 s), and **SC4** (640–960 s) with an incubation times of 5 minutes each. The graph at the bottom zooms in the nucleosome and water injection periods. In black is shown the stiffness response and in red the viscous losses responses. pH value of each sample is represented by the universal color code of pH paper and indicated in the inset (green for neutral pH, red for acidic pH). pH data from all samples are given in Table S1.

Then, after **SC4** injection on the reconstituted chromatin, the system is left during 5 minutes (Fig. 3A). A considerable mechanical effect was observed (Fig. 3C). The stiffness was detected as 15 N m^−1^ and the viscous losses as 2 × 10^−3^ N s m^−1^. Simultaneously, the DNA bundle between the SNT tip was observed using an optical inverted microscope, at 4X magnification. After the injection of **SC4**, the chromatin shape changes from a tight bundle to a wide web, but still strongly packed enough to be observable by optical microscopy, as shown in (Fig. 3B).

**Figure 4:**
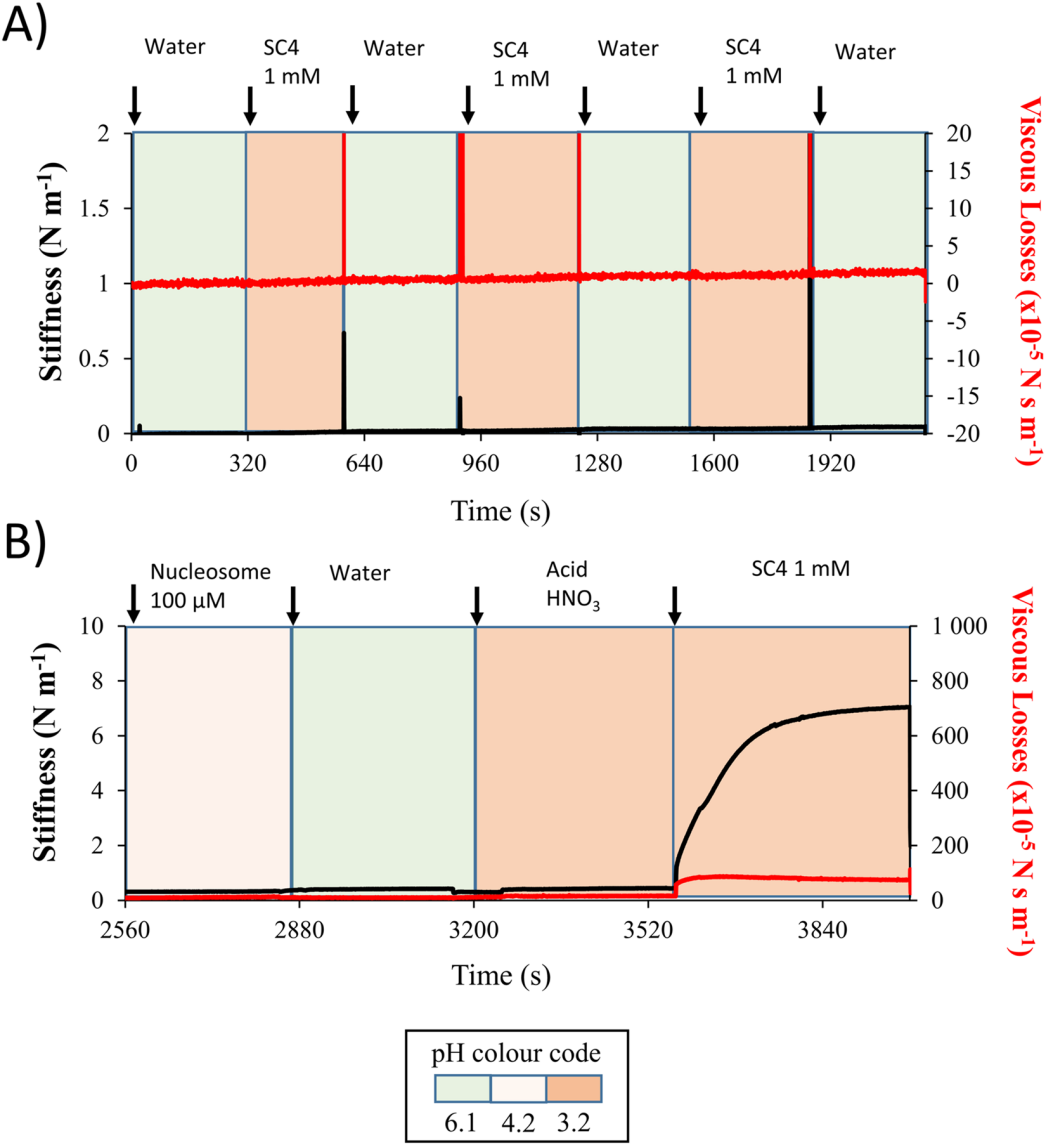
Mechanical effect of **SC4** on reconstituted chromatin through the specific interaction SC4/nucleosome. (**A**) Real time monitoring of the stiffness and viscous losses after 3 consecutive injections of water followed by 1 mM **SC4** on DNA, incubation times 5 minutes each. (**B**) Real time monitoring of the stiffness and viscous losses after chromatin reconstitution on SNTs, and then by the consecutives injections of water, Nitric Acid (pH 3) HNO_3_ followed by **SC4** on DNA, incubation times 5 minutes each. In black is shown the stiffness response and in red the viscous losses responses. Arrows indicate the moment of the injection. pH value of each sample is represented by the universal color code of pH paper and indicated in the inset (green for neutral pH, red for acidic pH). pH data from all samples are given in Table S1.

As a control experiment, **SC4** was injected in the channel to test the effect on DNA bundles but did not show any mechanical or physical effects (Fig. 4A and Supplementary Fig. S3). As expected, the negatively charged **SC4** has no affinity for the DNA polymer which carries a large number of negative phosphate ester groups. A second negative control experiment was performed by injecting a nitric acid solution having the same pH as that of the **SC4** solution, pH 3, on the reconstituted chromatin. The nitric acid solution resulted in a much smaller effect compared to the subsequent injection of **SC4** on the reconstituted chromatin showing once more a considerable mechanical effect (Fig. 4B). Finally, a third negative control experiment has been performed by monitoring the mechanical effect of *para*-hydoxybenzenesulfonic acid HBSA on the reconstituted chromatin (Supplementary Fig. S4A). HBSA possesses a phenolic unit and a sulphonate group as does SC4 with similar pKa but without being assembled in amacrocyclic. Unlike SC4, HBSA did not induce any increases in stiffness or viscous losses (Supplementary Fig. 4B,C). In a final control experiment a titration experiment was undertaken at 50 mM in Sodium Phosphate, this concentration was chosen to allow comparison with the work of Hof but more importantly to be at the Intracellular ionic concentration of Sodium^45^.

These negative control experiments confirm that these mechanical and physical effects are not induced by the acidity but specifically triggered by the complexation of the calix[4]arene derivative cavity toward the nucleosome complex. Hence, the interaction nucleosome/**SC4** induces a new organization of the reconstituted chromatin.

**Figure 5:**
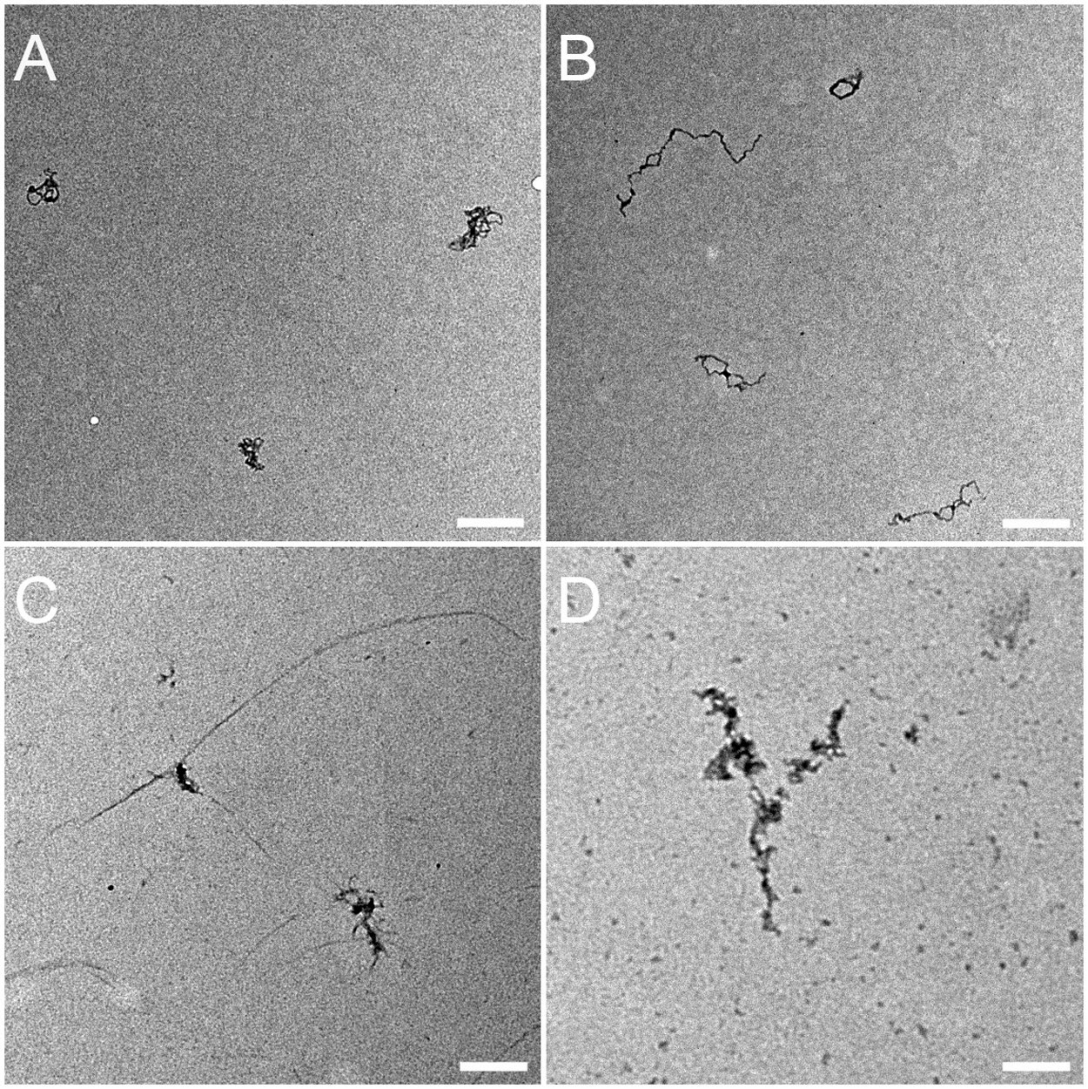
Effect of nucleosome and **SC4** on λ phage DNA in solution. TEM images of λ phage DNA (in **A**) mixed either with **SC4** (in **B**) or with nucleosome (in **C**) or nucleosome and **SC4** (in **D**). Scale bars are 200 nm.

**Figure 6:**
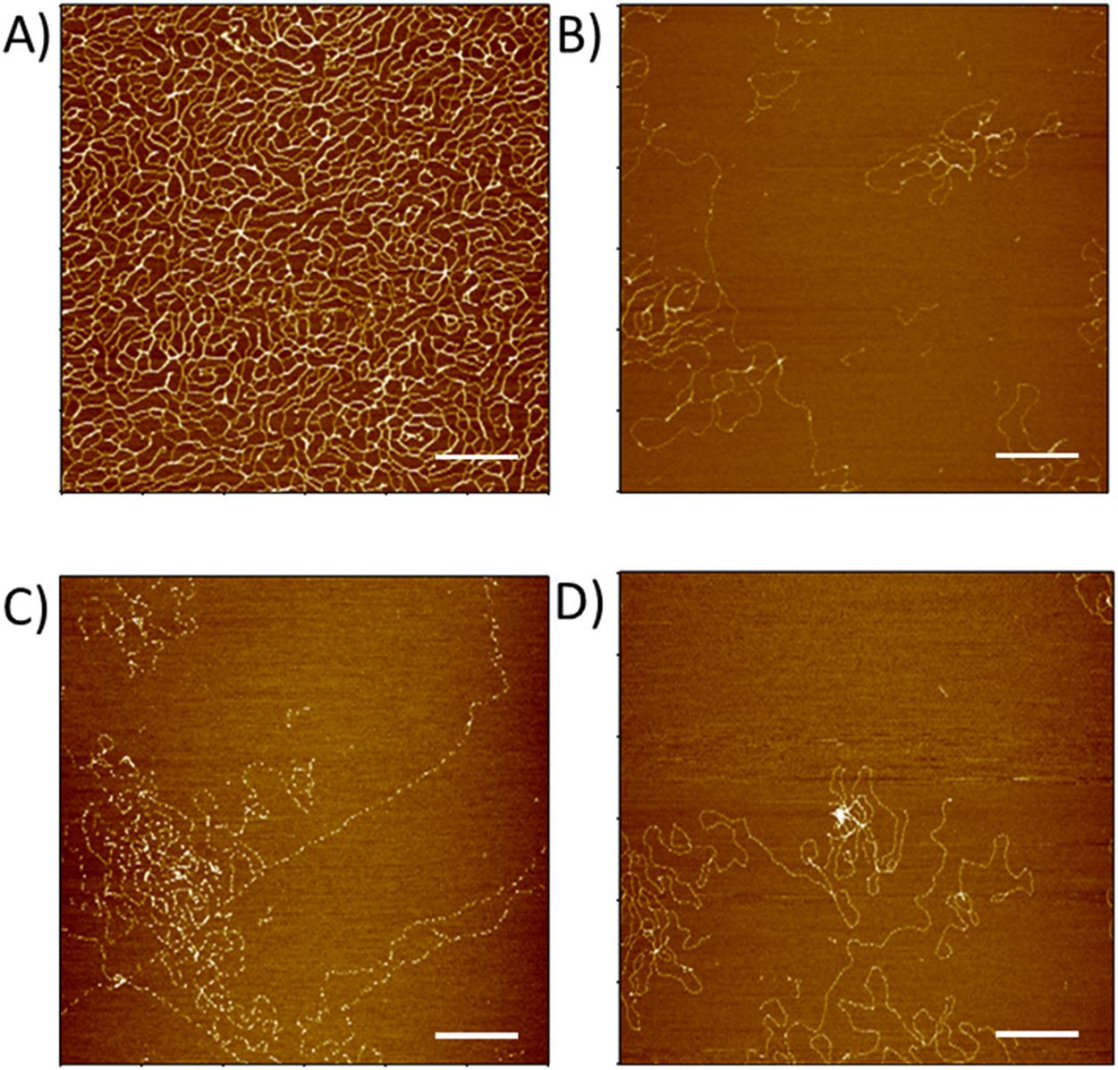
Effect of nucleosome and SC4 on λ phage DNA. AFM images of λ phage DNA (in **A**) mixed either with **SC4** (in **B**) or with nucleosome (in **C**) or nucleosome and **SC4** (in **D**). Scale bars are 500 nm.

**Figure 7:**
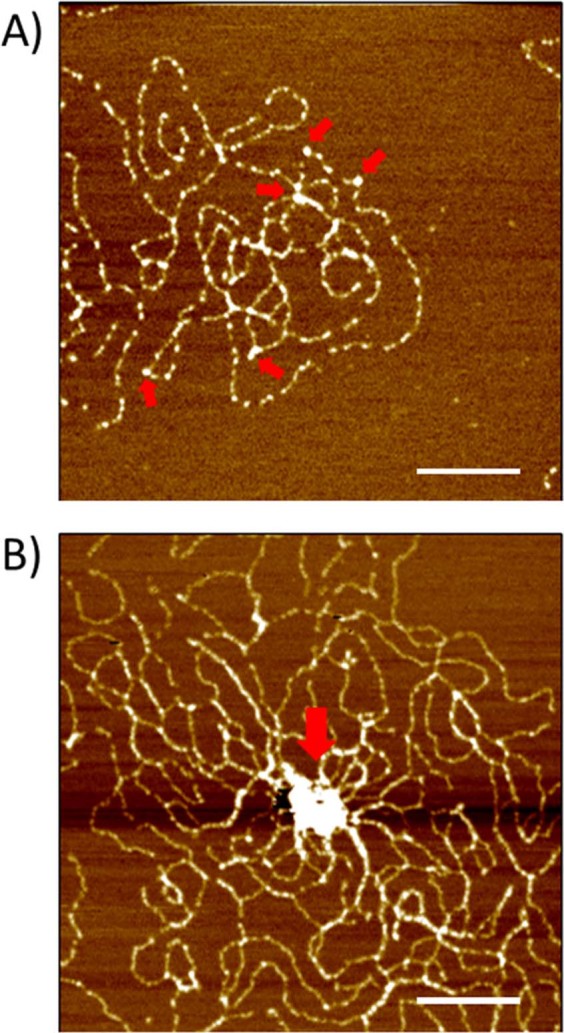
Effect of SC4 on nucleosome organisation along λ phage DNA. AFM images of λ phage DNA mixed either with nucleosome (in **A**) or nucleosome and **SC4** (in **B**). Scale bars are 200 nm. Red arrows indicate the presence of nucleosomes in A and a larger compact chromatin structure in B.

λ phage DNA mixed either with **SC4**, nucleosome, or the couple nucleosome/**SC4** in solution have been observed by Transmission Electronic Microscopy TEM (Fig. 5 and Supplementary Fig. S5) and Atomic Force Microscopy AFM (Fig. 6). Both methods show that when **SC4** is mixed with DNA, no or only a small loosening effect is observed on DNA arrangement (Figs 5B and 6B). This is in agreement with the absence of mechanical or optical change found with the SNT method. On the other hand, the DNA/nucleosome complex is clearly visible in AFM observations (Figs 6C and 7A) and shows a discrete assembly complex of 12–13 nm width along the DNA fiber which is in agreement with the characteristics of nucleosome assembly on DNA previously observed by AFM^43,46^. Height profiles of nucleosome are given in the Supplementary Information (Supplementary Figs S6–9. When SC4 is added to the reconstituted chromatin, larger structures are induced compared the nucleosome/DNA complex (Fig. 7B). These structures can be observed over larger surface at lower magnification (Supplementary Fig. S10) and present a size around 50 × 90 nm wide and 3 nm height (Supplementary Figs S11, 12. These microscopy observations support the optical observations on SNTs experiments in which **SC4** injection to DNA/nucleosome complex rearranges the chromatin organization.

## Discussion

The recognition abilities of *para*-sulphonato-calix[n]arene derivatives toward biomolecules and especially proteins, including histone proteins have been investigated in depth by the groups of Coleman^47,48^ and Hof^49^. These supramolecular host molecules possess a cage that can recognize the motif of these unstructured post translational modifications of cationic amino acids, which makes them ideal tools for probing epigenetic modifications of the nucleosome^50^. **SC4** has attracted special attention because it can make epigenetic modifications itself by preventing the interaction of specific post translational modifications reader enzymes on histone proteins in the nucleus of living cells^12^. The above shows that these supramolecular hosts are of great interest for therapeutic use as epigenetic Active Pharmaceutical Ingredients.

Here, for the first time we have used a new technique, mechanical characterization by SNTs, to easily reconstitute a chromatin and assess the dynamics and organization of such an assembly under the effect of this potential epigenetic Active Pharmaceutical Ingredient.

The reconstitution of the current chromatin analog in the microfluidic device is rapid taking about one hour as compared to the four days the is required using dialysis^29^. Moreover, the DNA compaction by nucleosomes can be confirmed by the real-time mechanical monitoring using SNTs. In the current method, the chromatin is assembled from a bundle having a DNA density estimated to be 0.8 × 10^6^–0.8 × 10^7^ bp µm^−3^ which is comparable to the DNA strands density of prokaryote (1.5 × 10^6^–1.5 × 10^7^ bp µm^−3^)^51^. Higher packing mode of chromatin in eukaryote cells can be reached only with nucleosome compaction^52^. It is then tempting to postulate that the reconstituted chromatin on SNTs might reach a similar packing level of DNA to that found in the nucleus of human cells (1 × 10^9^ bp µm^−3^). This bottom-up approach is thus very promising for reconstituting larger biomolecular complex on chromatin (enzymes, chemicals, Active Pharmaceutical Ingredients, etc) and for studying human biochemical processes.

In this study, the dynamics of a reconstituted chromatin under epigenetic modification reveals the considerable stiffening in presence of calix[4]arene **SC4**. SC4 has been already largely described for having a higher affinity toward amino acids bearing ammonium group such as lysine over other amino acids in water^17,47^. More recently, the group of Hof has characterized the complexation SC4/lysine on larger amino acid chain corresponding to the tail fragment of Histone H3 (Kd = 100 µM)^11^. At the high concentration of 1 mM, SC4 is then expected to interact with the lysine groups of histone protein. By neutralizing the lysine positive charges of histone, it should unpack the chromatin architecture. This type of neutralization has been already observed through the acetylation of histone protein H4. This post translational modification triggers the disruption of the inter-nucleosome interactions by neutralizing the lysine groups of the histone protein H4 (“basic patch”) and releasing them from the “acid patch” of the histone protein dimer, H2AH2B present in the adjacent nucleosome^53^. As a consequence, the chromatin is loosened in its structure^54^ and possibly let DNA molecules to push each other away to enlarge the bundle cross-sectional area. This mechanism proposal is supported by the change of chromatin organization at the tweezers tips upon the addition of **SC4** with strands that visually appear longer (Fig. 3). This reorganization of chromatin may lead the non-bridging DNA molecules at SNT arms becoming involved with the overall structure. The asymmetric geometry of formed web structure suggests that the flow created during SC4 insertion (from left to right of the observation area; Fig. 3B) help the initially unbound DNA to be included in the newly formed web structure. Therefore, any effect we could see in the chromatin was amplified and resulted in a dramatic change of stiffness and viscous losses.

This change in the chromatin organization will be expected to modify not only the interactions of other proteins that interact with histone, such as MLL5 plant homeodomain finger protein readers identified recently^12^, but also to affect gene regulation and the subsequent cellular activities by changing gene accessibility. An extensive biological study remains to be carried out to understand fully the epigenetic effects of such a molecule on gene expression and phenotype.

## Conclusion

Here, through the simple study case of the *para*-sulphonato-calix[4]arene on chromatin, we have demonstrated how a method of mechanical characterization can prove to be extremely useful in assessing the effects of a potential epigenetic Active Pharmaceutical Ingredient on the dynamics of chromatin.

In terms of the wide potential applications in epigenetics, this is a fast, cost effective and practical method for reconstituting a biomolecular complex in a crowded environment and for identifying mechanical and physical effects of epigenetic modifications although the DNA capturing process of the proposed protocol should be improved to obtain pre-defined and targeted amount of bridging DNA consistently.

This technique enables the study of a wide concentration range of bio-active molecules on DNA. This is of great importance to determine the optimal concentration at which the chromatin is reorganized. Again, the example of **SC4** is very illustrative because it possesses a higher affinity for the tri methylated lysine over other post translational modifications of lysine groups of histone proteins^55^.

Finally, this new method offers an innovative tool to researchers wishing to better examine the dynamics of chromatin in predicting or modifying the cellular activities and its subsequent physiological effects.

This proof-of-concept study can be extended to other biochemical reactions that occur on chromatin under appropriated salts concentrations and buffers which fit better to the nucleus environment.

## Methods

### Materials

All chemicals were purchased from ACROS Organics or Sigma Aldrich and used without further purification. Solvents were of chemical grade and were used without any purification. Water was obtained on a System Direct-Q 3 Reverse Osmosis Purifier (Merck, Japan) and used with a resistivity of 18 MOhm. All histone protein types and nucleosome have been purified from calf thymus and supplied in lyophilized powder. Histone protein H2A (reference H9250), Histone protein H3 (reference 11034758001) and nucleosome containing a mixture of histone proteins H1, H2A, H2B, H3, and H4 (reference 10223565001) have been purchased from Sigma Aldrich. The histone protein duplex H3/H4 (Reference MBS634742) has been purchased from the MyBioSource company. Lyophilized histone proteins and nucleosome samples were re-suspended in aqueous solution. The pH of sample solution was measured using a compact pH meter, twin pH (Horiba, Japan). A table reporting the pH values is given in the Supplementary Data (Supplementary Table S1).

### Synthesis and characterization of *para*-sulphonato calix[4]arene

*Para*-sulphonato-calix[4]arene **SC4** has been synthesized following the literature method for biological applications of *para*-sulphonato-calix[n]arenes^56^. All the physical characteristics are in agreement with the literature values.

### Real-time measurements with SNT

The details of real-time measurements with SNT^36^ and the use of titration experiments for determining the mechanical properties of DNA^39^ have been previously described.

The amount of captured DNA between SNT tips differs within a 10-fold range based on the quality of the aluminium layer on the tips and the gap between the tips. The vast majority of the bundles contained 400–5000 DNA molecules between the tips. This number was calculated using the change in the detected stiffness values by the SNT with and without the DNA bundle in a solution (corresponding to the bundle stiffness) and divided by the stiffness of a single DNA molecule in a similar solution available in the literature^57,58^.

In the experiments, firstly the mechanical effect on λ phage DNA (Takara-Bio) of histone H2A (increasing concentrations from 500 nM to 50 µM), histone protein H3 (increasing concentrations from 0.1 nM to 1 µM), and histone protein duplex H3/H4 (increasing concentrations from 0.1 nM to 1 µM) were assessed. In a second time the mechanical effects of nucleosome solutions (increasing concentrations from 0.1 nM to 100 nM) were measured by titration experiments. Purified calf thymus histone types and nucleosome were purchased from Sigma Aldrich and York Bio companies. All experiments were carried out in duplicate on separate samples of DNA.

For the examination of the mechanical effects of SC4, a reconstituted chromatin was formed by injecting a concentration range of nucleosome (increasing concentrations from 0.1 nM to 100 µM) onto the suspended DNA bundle, which was washed with water, via the micro-fluidic system, and finally titrated with SC4 at 1 mM.

In all cases firstly, the captured DNA bundle was inserted in the liquid, at the aperture of the fluidic system. Then for or each protein solution, the protein was introduced during 20 seconds of a flow period, followed by 5 min of incubation time and then, another 20 seconds of flow of the following solution to change the solution in contact with the DNA bundle. In all cases, an increasing concentration step gradient was used.

### Optical Microscopy

Optical imaging was carried out using an inverted optical microscope (Olympus, IX71) with the SNT set-up installed on the sample stage.

### TEM observation

All samples were fixed with 0.1% of Glutaraldehyde TEM grade (Sigma Aldrich), washed with water, stained with 2% of filtered Uranyl acetate and finally washed with water.

All treated samples were deposited on a formvar carbon coated grid previously activated by plasma. Observation was carried out using a Philips CM120 TEM at 80 kV.

### AFM observation

All samples were fixed with 0.1% of Glutaraldehyde TEM grade (Sigma Aldrich) and prepared as the method given by Gerling Cervantes for observing individual λ-DNA phage by AFM^59^ with some slight modifications. Samples were deposited with 10 mM of MgCl_2_ onto a freshly cleaved mica previously mounted on a glass slide. The sample was left 2 minutes and spun with a spin coating system Mikasa 1H-D7 (Mikasa Co Ltd, Tokyo, Japan) for 20 seconds at 500 rpm and for 20 seconds at 7000 rpm. The unbound biomolecules were removed from mica surface by depositing a droplet of water and spun for 20 seconds at 500 rpm and for 2 minutes at 7000 rpm.

AFM observations were performed on a MFP-3D Infinity Asylum Research (Oxford Instruments, Tokyo, Japan) operated in tapping mode, using a silicon micro cantilever tip (Olympus, AC200TS-RS) with a height of 14 µm and a radius of 7 nm, a spring constant of 9 N/m and a resonance frequency of 180 kHz. Line scan rates were 0.5–1 Hz and images were acquired at 512 × 512 pixels.

## Supplementary information

Supplementary Informations

## Data Availability

The datasets generated during and/or analysed during the current study are available from the corresponding author on reasonable request.
