# Supplementary material for: Direct measurement of the mechanical properties of a chromatin analog and the epigenetic effects of para-sulphonato-calix[4]arene
Source: Sci Rep. 2019 Apr 9;9:5816. doi: 10.1038/s41598-019-42267-x (PMC6456576; doi:10.1038/s41598-019-42267-x)
Supplement: Supplementary file 1 — Supplementary Informations [file 41598_2019_42267_MOESM1_ESM.pdf]

Direct measurement of the mechanical properties of a chromatin analog and the epigenetic effects of *para*-sulphonato-calix[4]arene

*Yannick Tauran[1,2 *], Momoko Kumemura[2,3,4], Mehmet C. Tarhan[5,6], Grégoire Perret[2,6], Florent Perret[7], Laurent Jalabert[2,3], Dominique Collard[2,6], Hiroyuki Fujita[2,3], and Anthony W. Coleman FRSC[3]*

[1] LMI CNRS UMR 5615, Université Lyon 1, Villeurbanne, 69622, France;
[2] LIMMS/CNRS-IIS UMI 2820, Institute of Industrial Science, The University of Tokyo, Tokyo, 153-8505, Japan;
[3] CIRMM, Institute of Industrial Science, The University of Tokyo, Tokyo, 153-8505, Japan;
[4] Graduate School of Life Science and Systems Engineering, Kyushu Institute of Technology, Fukuoka, 808-0196, Japan;
[5] Univ. Lille, CNRS, Centrale Lille, ISEN, Univ. Valenciennes, UMR 8520 - IEMN, Lille, F59000, France;
[6] CNRS/IIS/COL/Lille 1 SMMiL-E project, 59046, Lille Cedex, France;
[7] ICBMS, CNRS UMR 5246, Université Lyon 1, Villeurbanne, 69622, France.

* Yannick Tauran
LMI CNRS UMR5615, Université Lyon 1,
Villeurbanne, 69622, France
Tel: + 33 4 7243 2962
yannick.tauran@univ-lyon1.fr

Supplementary Figures and Table

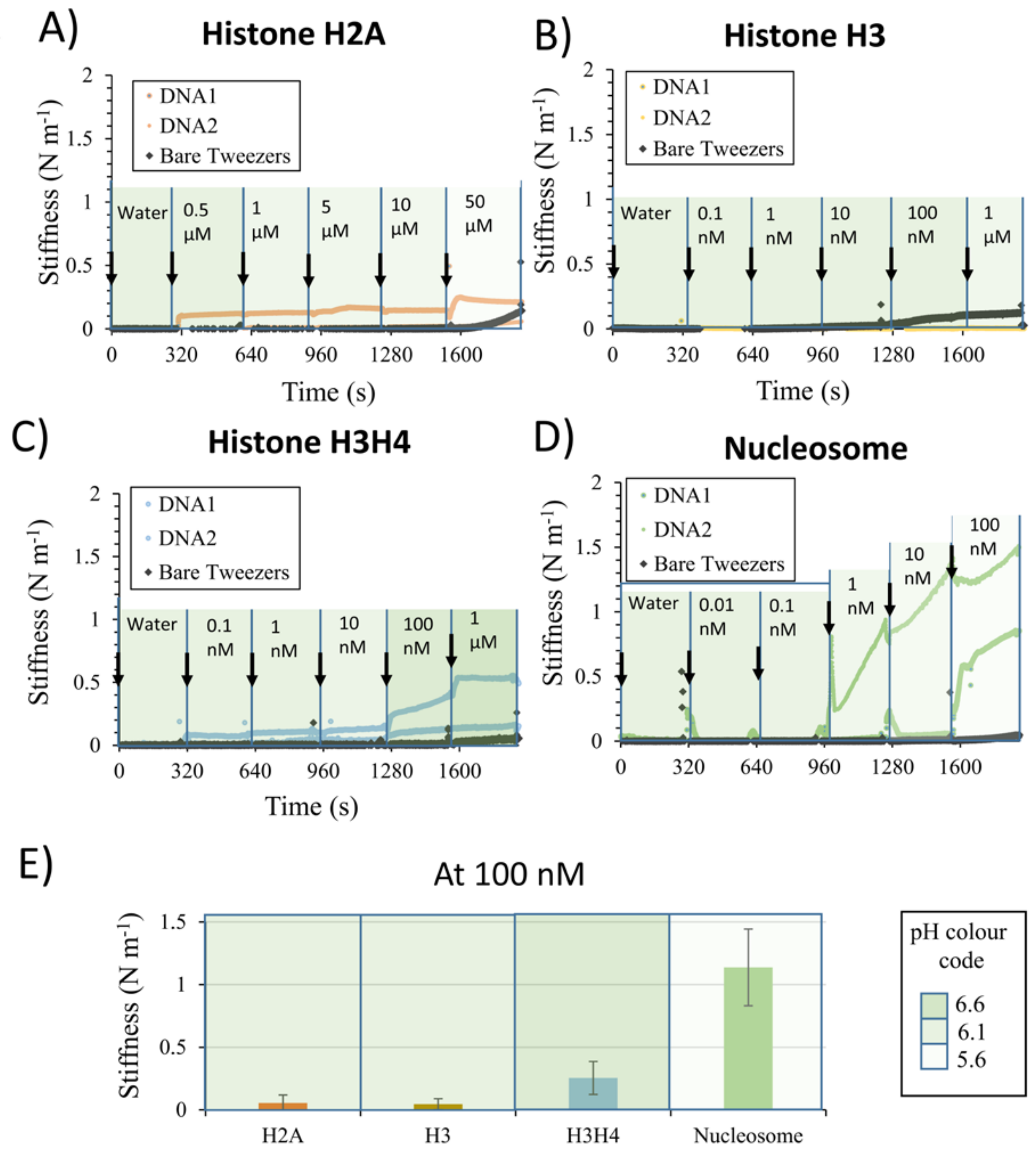

Figure S1 – Stiffening effect on DNA of nucleosome compared to histones types for reconstituting chromatin. Real time monitoring of the stiffness on SNTs by injecting increasing concentration of (A) histone protein H2A, (B) histone protein H3, (C) histone protein duplex H3/H4, and (D) nucleosome on two different captured DNA. (E) Histogram of stiffness averaged on two captured DNA, after 4 minutes of incubation of either histone protein H2A, histone protein H3, histone protein duplex H3/H4, or nucleosome at 100 nM. pH value of each sample is represented by the universal color code of pH paper and indicated in the inset (green for neutral pH, red for acidic pH). pH data from all samples are given in Table S1.

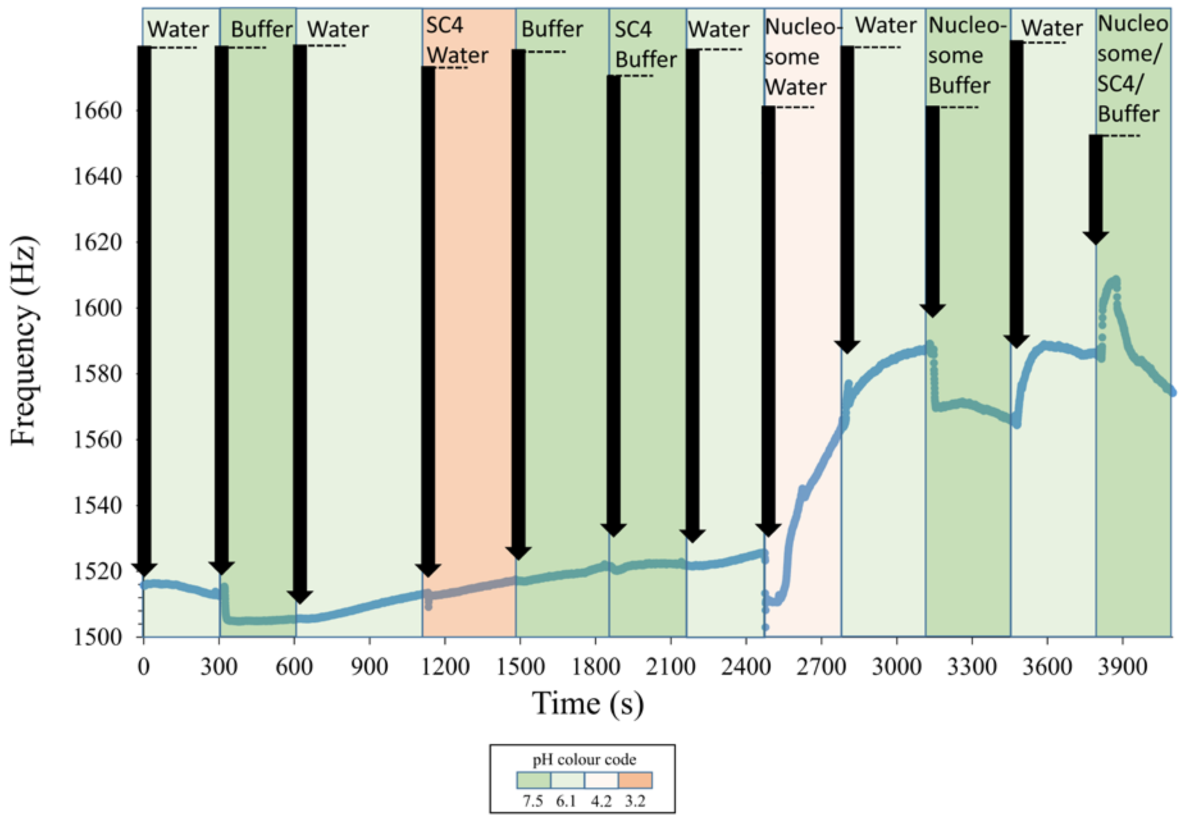

Figure S2 – Real time monitoring of stiffening effect of nucleosome and calix[n]arene SC4 on DNA under water and 50 mM sodium phosphate, pH 7.4 buffer conditions. Real time monitoring by SNTs of the change of the resonance frequency of DNA under consecutive injection of water, buffer, water, SC4 in water, buffer, SC4 in buffer, water, nucleosome in water, water, nucleosome in buffer, water, nucleosome and SC4 in buffer and water. Black arrows indicate the moment of the injection of each sample. Samples were then incubated for at least 300s with DNA. Blue color represents sample diluted in water or water only, while Light orange color represents sample diluted buffer or buffer only. pH value of each sample is represented by the universal color code of pH paper and indicated in the inset (green for neutral pH, red for acidic pH). pH data from all samples are given in Table S1.

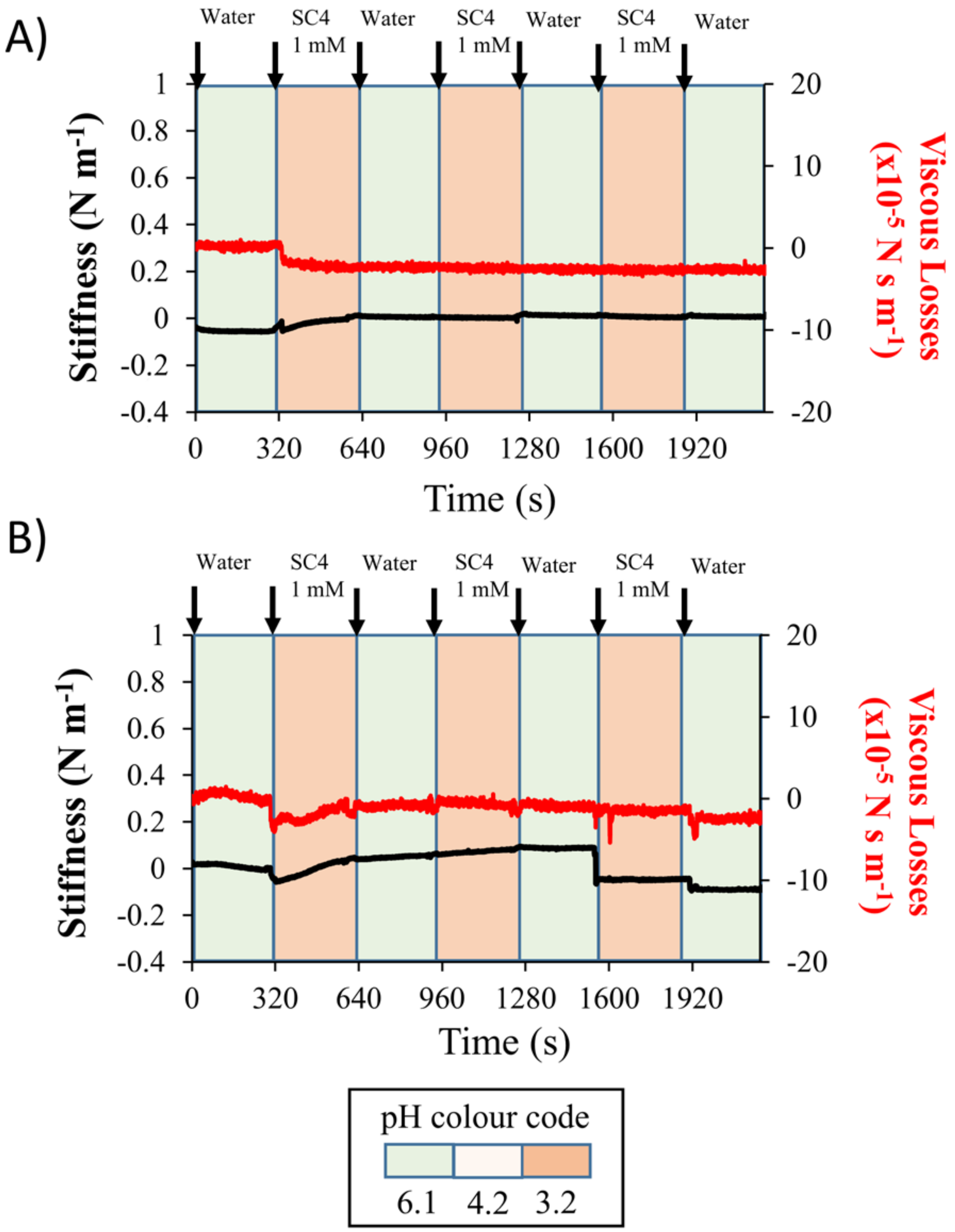

Figure S3 – No observable mechanical effects when SC4 is mixed directly on DNA. Real time monitoring of the stiffness and viscous losses after 3 consecutive injections of water followed by **SC4** on 2 different captured DNA (A and B). Incubation times 5 minutes each. pH value of each sample is represented by the universal color code of pH paper and indicated in the inset (green for neutral pH, red for acidic pH). pH data from all samples are given in Table S1.

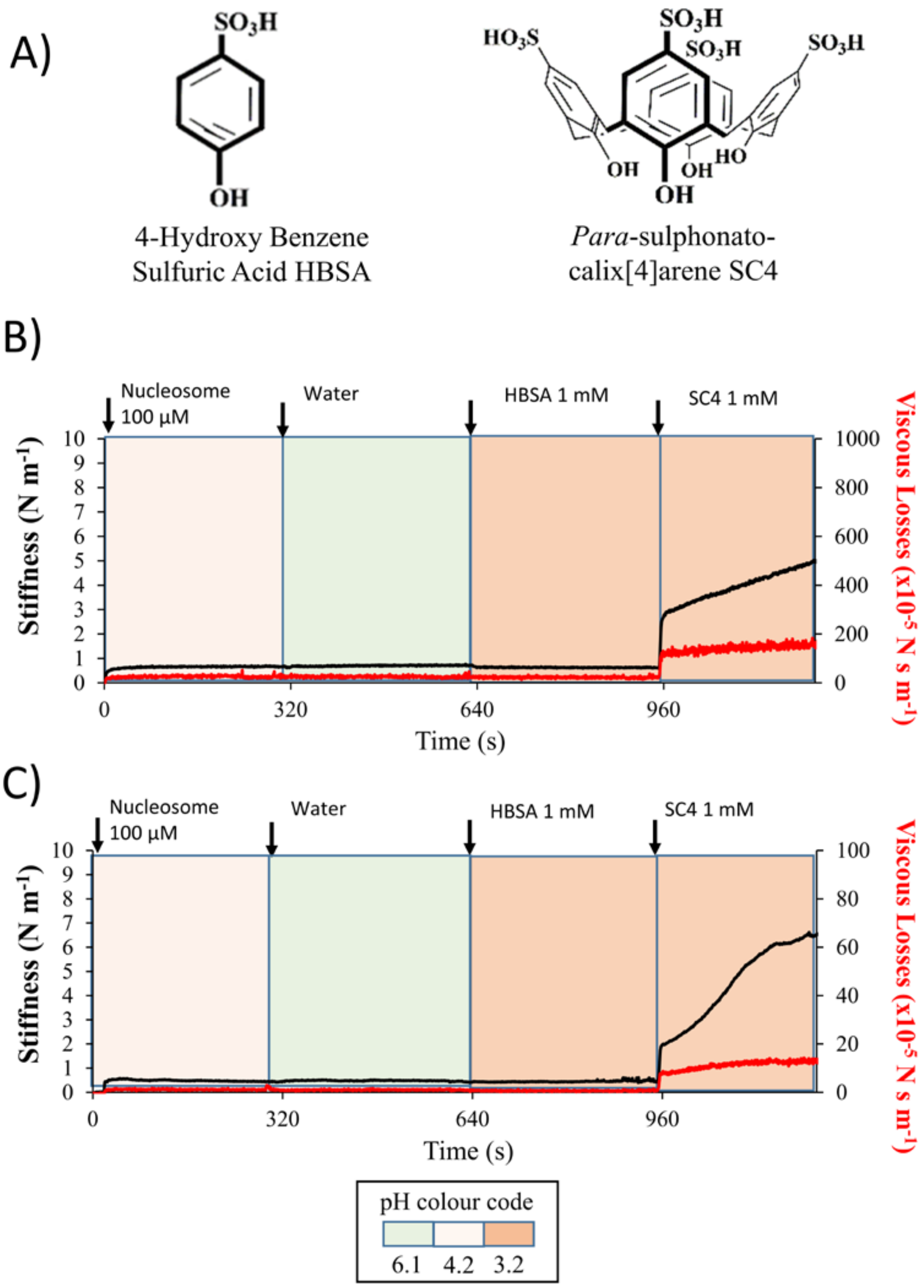

Figure S4 – Mechanical effect induced by SC4 cavity complexation toward the reconstituted chromatin. (A) Structures of 4-Hydroxy Benzene Sulfuric Acid HBSA (on the left) and Para-sulphonato-calix[4]arene SC4 (on the right). (B-C) Duplicates of real time monitoring of the stiffness and viscous losses after chromatin reconstitution on SNTs by 100 µM of nucleosome incubation, and then by the consecutives injections of water, 1 mM of **HBSA** followed by 1 mM of **SC4** on DNA, incubation times 5 minutes each. In black is shown the stiffness response and in red the viscous losses responses. Arrows indicate the moment of the injection. pH value of each sample is represented by the universal color code of pH paper and indicated in the inset (green for neutral pH, red for acidic pH). pH data from all samples are given in Table S1.

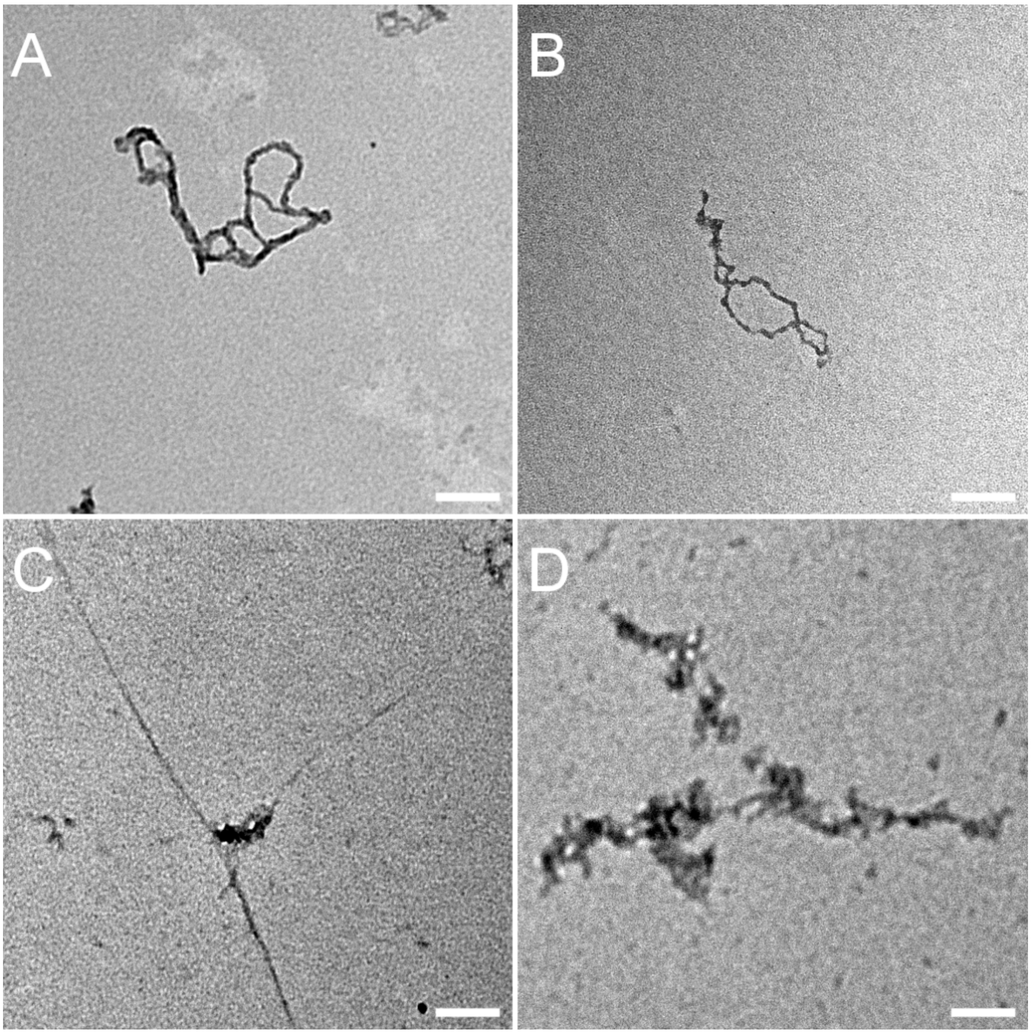

Figure S5 – Effect of nucleosome and SC4 on λ phage DNA. TEM images of λ DNA (in A) mixed either with **SC4** (in B) or with nucleosome (in C) or nucleosome and **SC4** (in D). Bar scale is 50 nm.

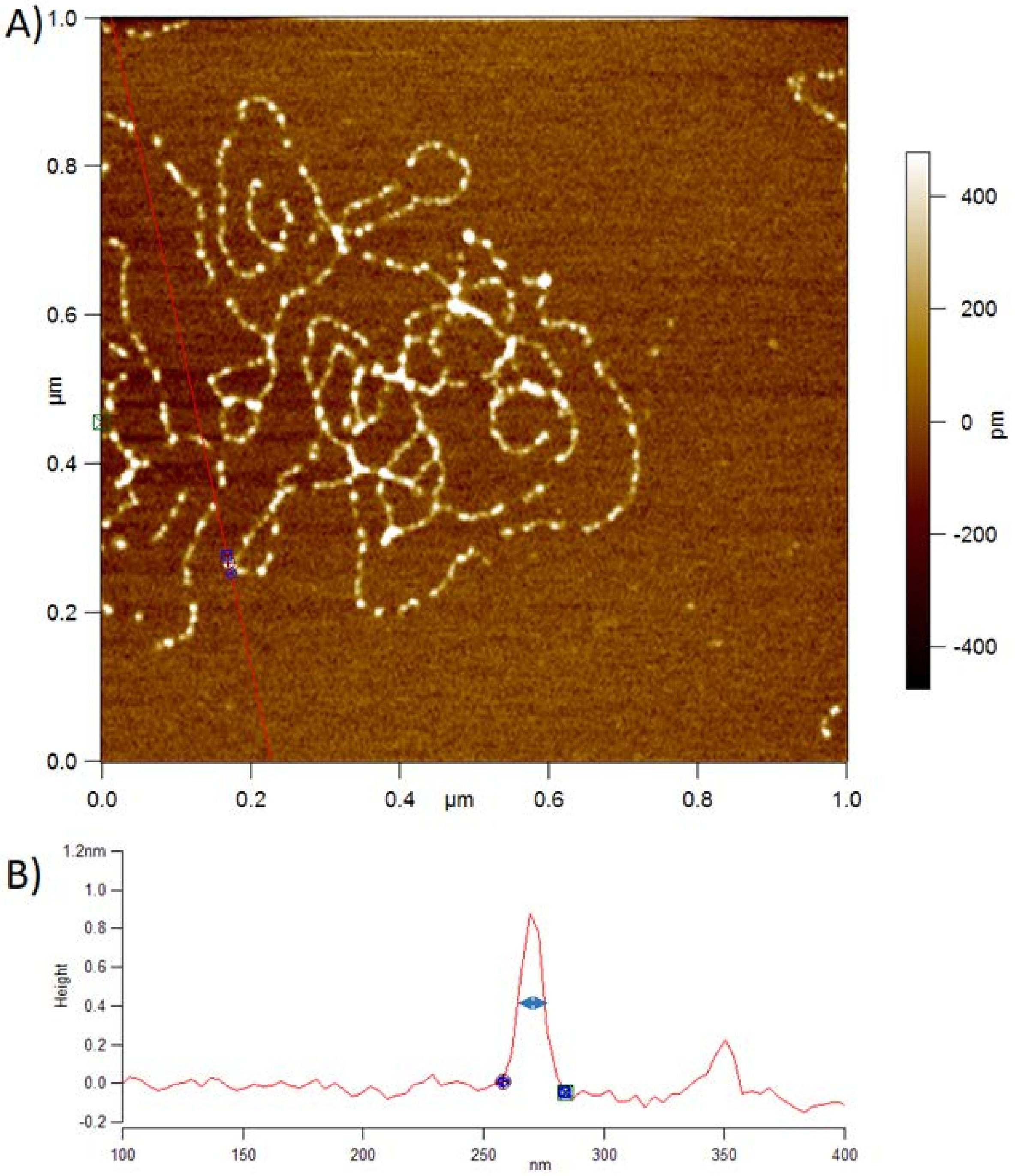

Figure S6. Size analysis of nucleosome on λ phage DNA. (A) AFM images of λ phage DNA 1 x 1 µm^2. (B) Height profile of the transversal section of a nucleosome marked with a red line in A and bordered by 2 blue plots. Blue line with double arrows at half height indicates a size of nucleosome of 12 nm wide and 0.9 nm height*.

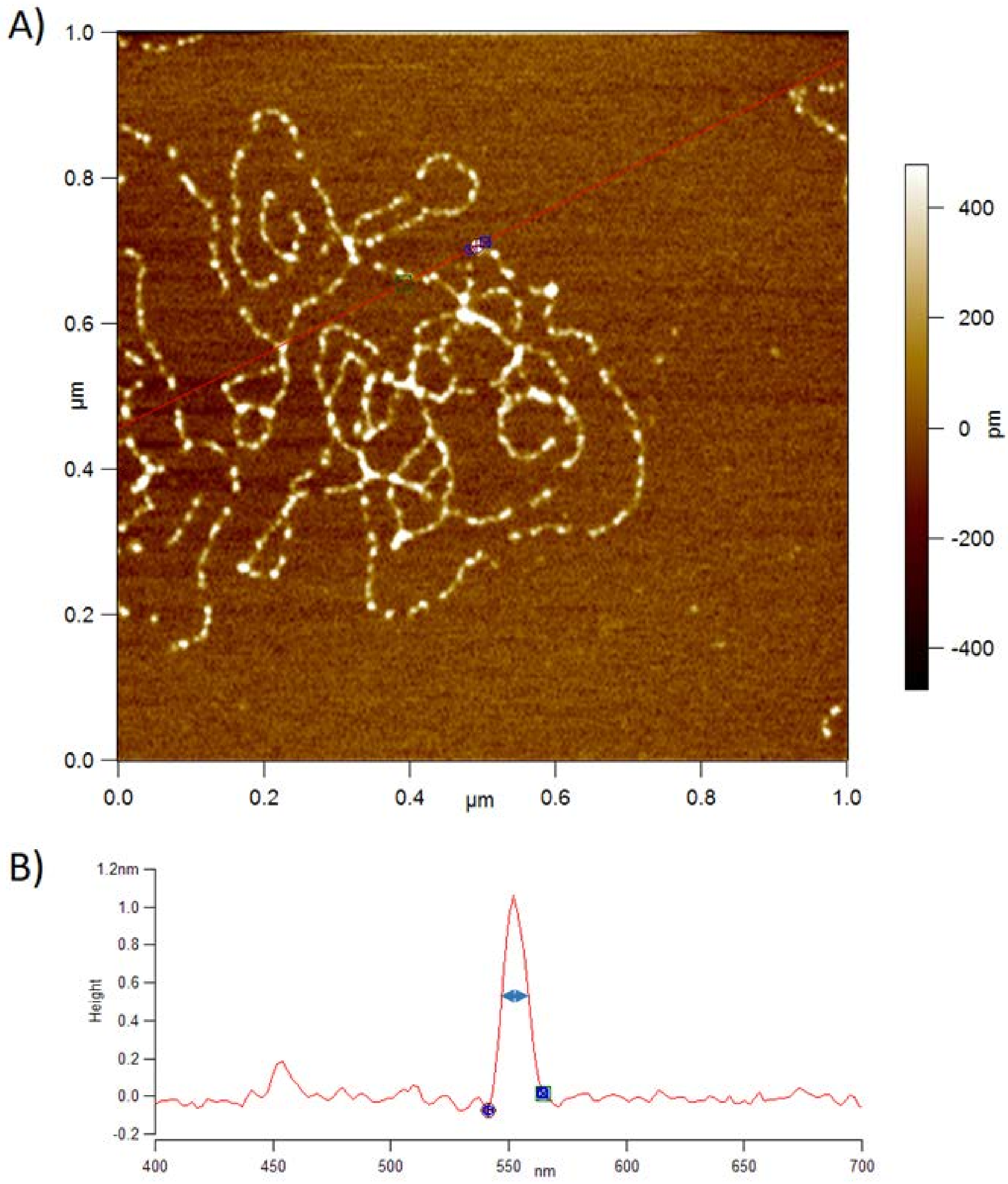

Figure S7. Size analysis of nucleosome on λ phage DNA. (A) AFM images of λ phage DNA 1 x 1 μm^2. (B) Height profile of the transversal section of a nucleosome marked with a red line in A and bordered by 2 blue plots. Blue line with double arrows at half height indicates a size of nucleosome of 12 nm wide and 1 nm height*.

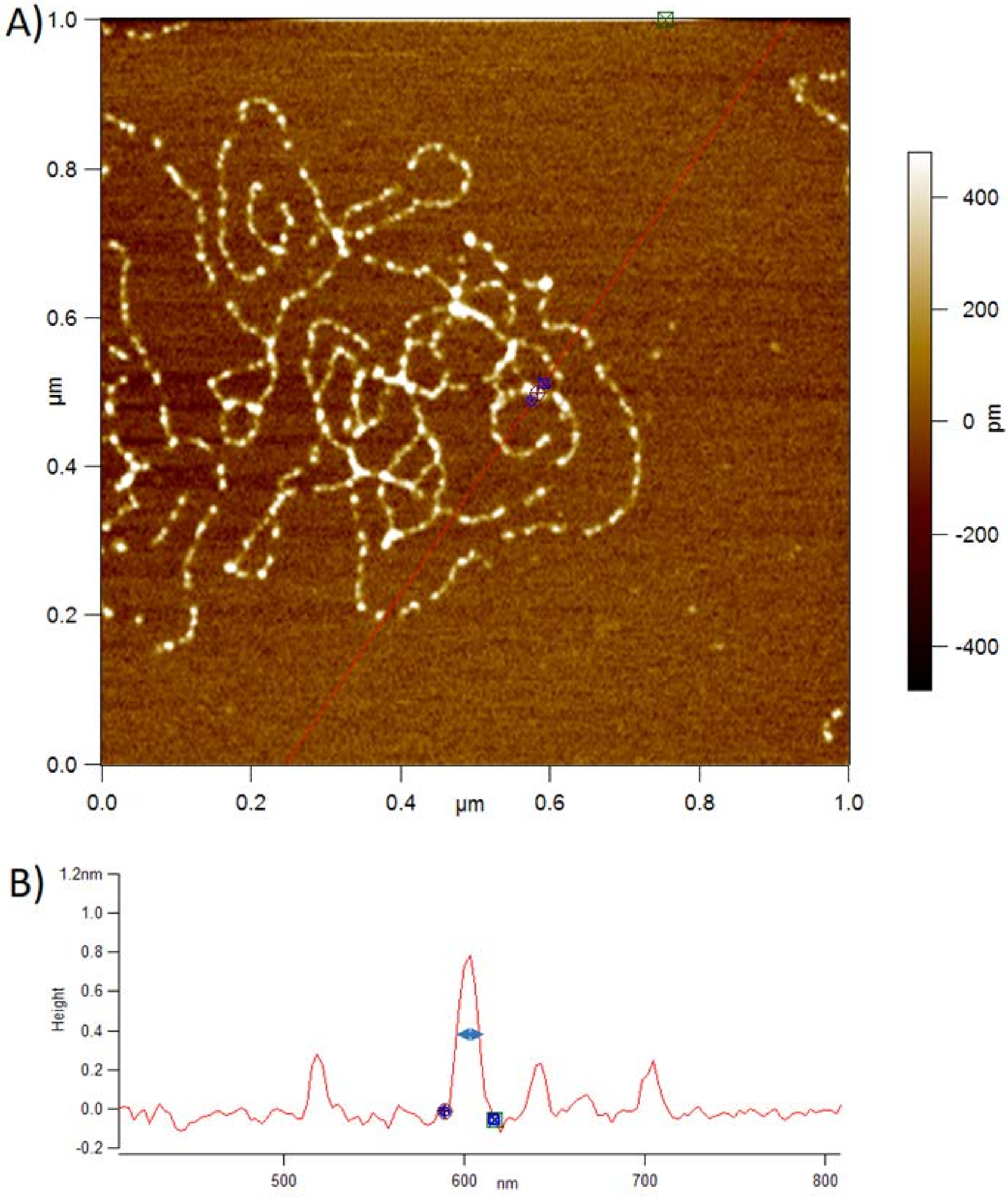

Figure S8. Size analysis of nucleosome on λ phage DNA. (A) AFM images of λ phage DNA 1 x 1 µm^2. (B) Height profile of the transversal section of a nucleosome marked with a red line in A and bordered by 2 blue plots. Blue line with double arrows at half height indicates a size of nucleosome of 12,1 nm wide and 0.7 nm height*.

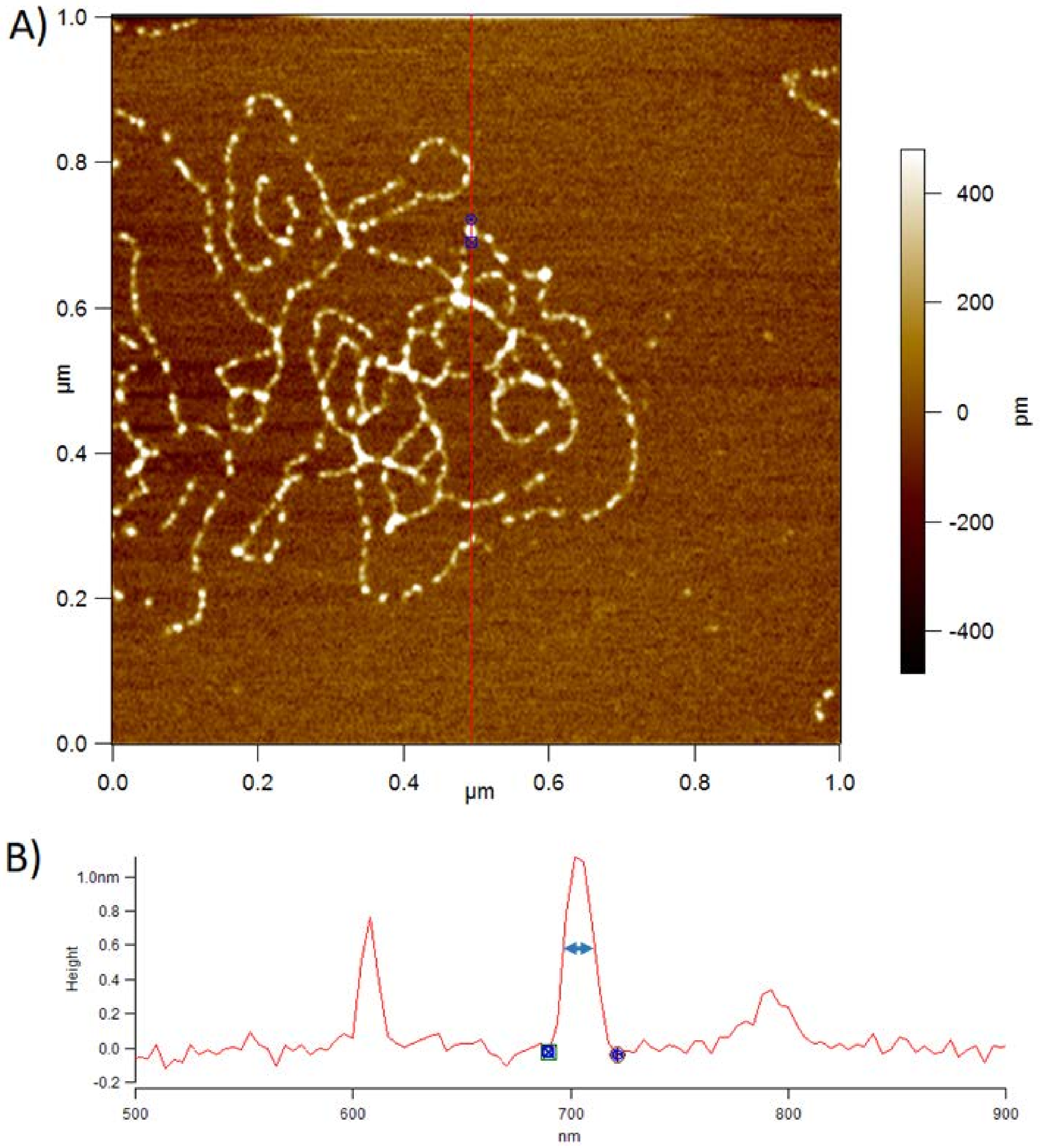

Figure S9. Size analysis of nucleosome on λ phage DNA. (A) AFM images of λ phage DNA 1 x 1 µm^2. (B) Height profile of the transversal section of a nucleosome marked with a red line in A and bordered by 2 blue plots. Blue line with double arrows at half height indicates a size of nucleosome complex of 13.7 wide and 1 nm height*.

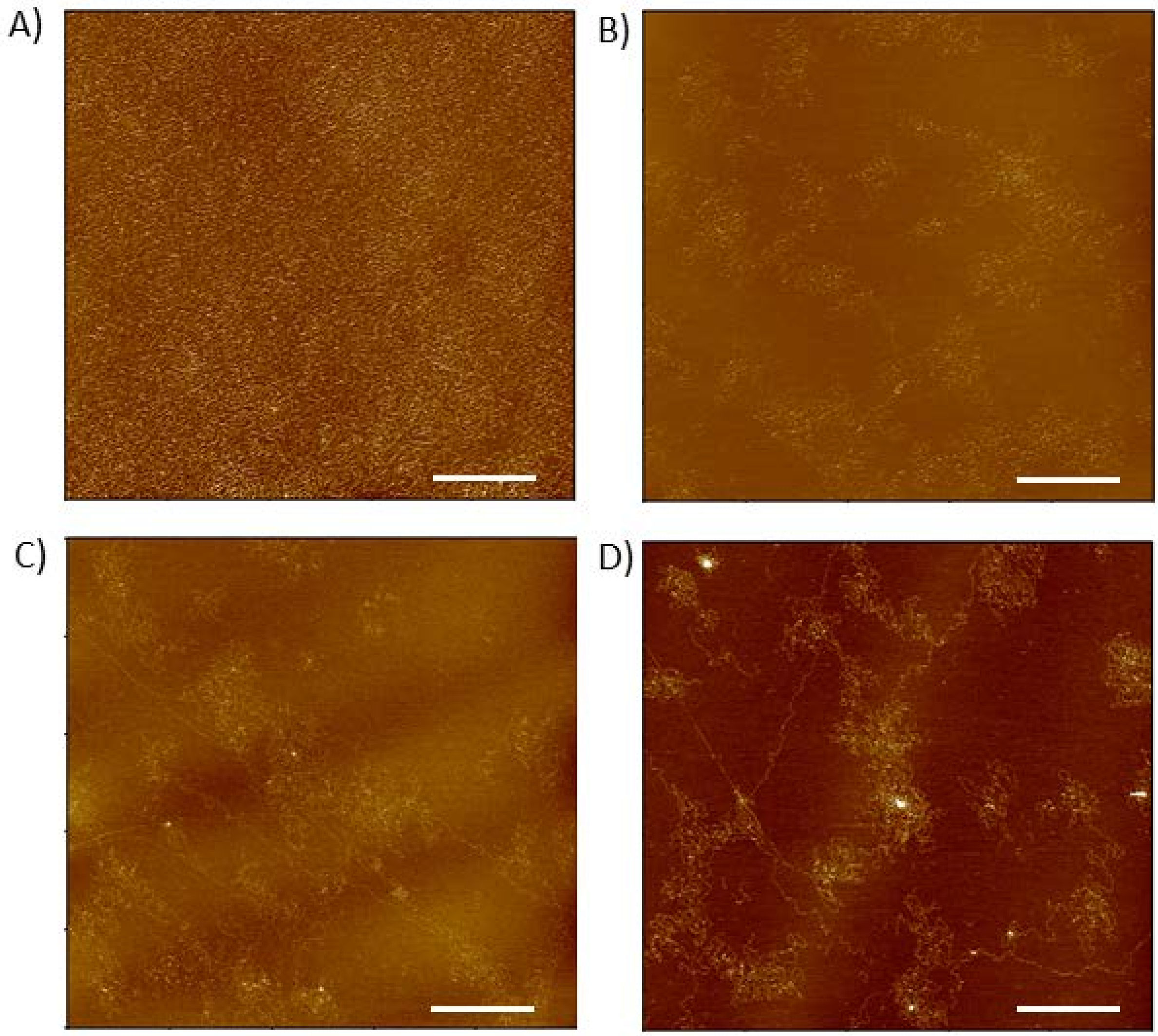

Figure S10. Effect of nucleosome and SC4 on λ phage DNA. AFM images of λ phage DNA (in A) mixed either with **SC4** (in B) or with nucleosome (in C) or nucleosome and **SC4** (in D). Scale bars are 2 µm.

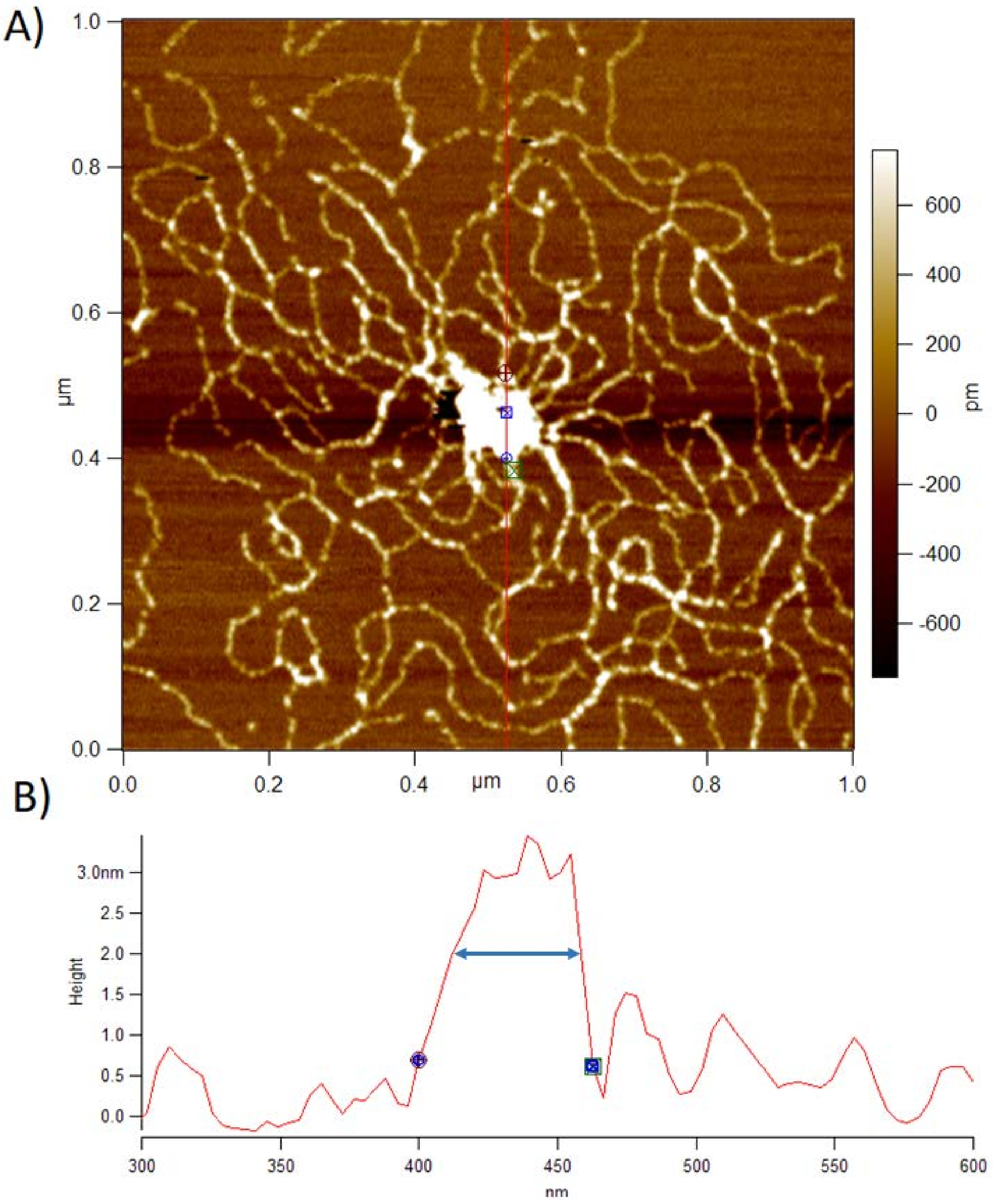

Figure S11. Size analysis of nucleosome/SC4 complex on λ phage DNA. (A) AFM images of λ phage DNA 1 x 1 µm^2. (B) Height profile of the transversal section of the nucleosome/SC4 complex marked with a red line in A and bordered by 2 blue plots. Blue line with double arrows at half height indicates a size of the complex of 48 nm wide and 3 nm height*.

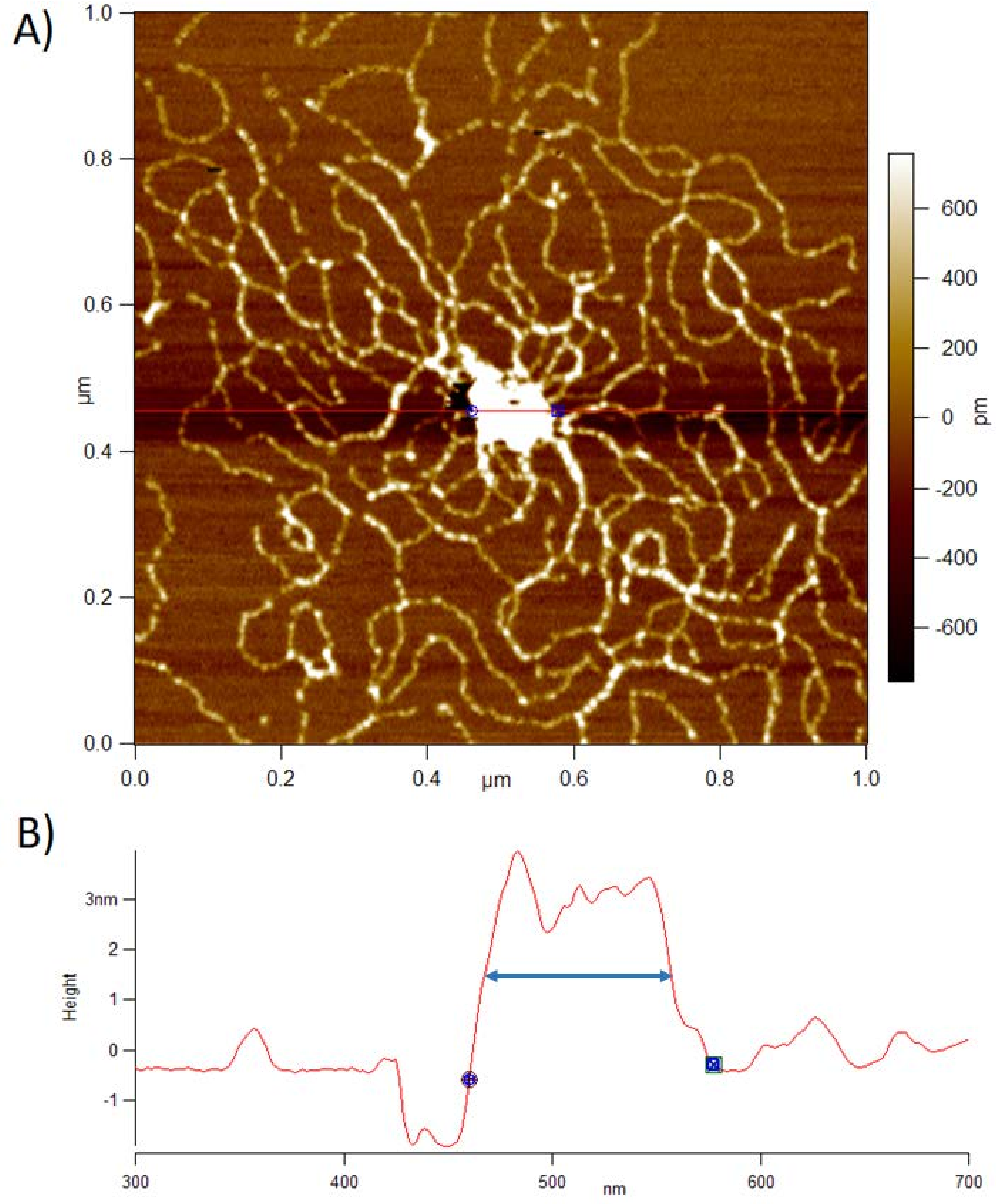

Figure S12. Size analysis of nucleosome/SC4 complex on λ phage DNA. (A) AFM images of λ phage DNA 1 x 1 μm^2. (B) Height profile of the transversal section of the nucleosome/SC4 complex marked with a red line in A and bordered by 2 blue plots. Blue line with double arrows at half height indicates a size of the complex of 90 nm wide and 3 nm height*.

Table S1. pH values of sample solutions.

Compounds	pH
DI Water	6.1
SC4 1 mM	3.2
para-hydroxybenzene sulfonic acid 1 mM	3.2
Nitric acid 1 mM	3.1
Histone H2A 50 µM	5.5
Histone H2A 10 µM	5.5
Histone H2A 5 µM	5.6
Histone H2A 1 µM	5.8
Histone H2A 500 nM	6
**Histone H2A 100 nM**	**6.1**
Histone H3 1 µM	5.7
**Histone H3 100 nM**	**6.1**
Histone H3 10 nM	6.1
Histone H3 1 nM	6.1
Histone H3 0.1 nM	6.1
Histone H3H4 1 µM	6.6
**Histone H3H4 100 nM**	**6.3**
Histone H3H4 10 nM	6.2
Histone H3H4 1 nM	6.1
Histone H3H4 0.1 nM	6.1
Nucleosome 100 µM	4.2
Nucleosome 1 µM	5
**Nucleosome 100 nM**	**5.6**
Nucleosome 10 nM	5.7
Nucleosome 1 nM	5.8
Nucleosome 0.1 nM	6
Nucleosome 0.01 nM	6.1
λ phage DNA at 0.175 mg mL^{-1}	7.5
λ phage DNA at 0.175 mg mL-1/ Nucleosome 100 µM	5.8
λ phage DNA at 0.175 mg mL-1 / Nucleosome 100 µM / SC4 1 mM	3.9
λ phage DNA at 0.175 mg mL-1 / SC4 1 mM	3.8
SC4 1 mM	3.2
SC4 100 µM	3.8
SC4 10 µM	4.4
SC4 1 µM	5.3

Note and reference

*AFM height gave lower measurements than expected because of soft structures [1]

1 Dubes A., Parrot-Lopez H., Abdelwahed W., Degobert G., Fessi, H., Shahgaldian, P., Coleman A.W. Scanning electron microscopy and atomic force microscopy imaging of solid lipid nanoparticles derived from amphiphilic cyclodextrins. *European Journal of Pharmaceutics and Biopharmaceutics* **55**(3), 279-282 (2003).
